# Development and characterization of the novel human osteosarcoma cell line COS-33 with sustained activation of the mTOR pathway

**DOI:** 10.18632/oncotarget.27611

**Published:** 2020-07-07

**Authors:** Ashley VanCleave, Mykayla Palmer, Fang Fang, Haydee Torres, Tania Rodezno, Qilin Li, Kirby Fuglsby, Claire Evans, Yohannes Afeworki, Alan Ross, Pulivarthi Rao, Patricia Leiferman, Siyuan Zheng, Peter Houghton, Jianning Tao

**Affiliations:** ^1^Cancer Biology and Immunotherapies Group, Sanford Research, Sioux Falls, SD, USA; ^2^SPUR Scholar Program, University of South Dakota, Sioux Falls, SD, USA; ^3^Department of Chemistry and Biochemistry, South Dakota State University, Brookings, SD, USA; ^4^Greehey Children’s Cancer Research Institute, University of Texas Health Science Center at San Antonio, San Antonio, TX, USA; ^5^Department of Biomedical Engineering, University of South Dakota, Sioux Falls, SD, USA; ^6^Functional Genomics & Bioinformatics Core Facility, Sanford Research, Sioux Falls, SD, USA; ^7^Sanford Medical Genetics Laboratory of Sanford Health, Sioux Falls, SD, USA; ^8^Texas Children’s Cancer and Hematology Centers, Department of Pediatrics, Baylor College of Medicine, Houston, TX, USA; ^9^EGL Genetics Laboratory, Tucker, GA, USA; ^10^Department of Pediatrics, Sanford School of Medicine, University of South Dakota, Sioux Falls, SD, USA

**Keywords:** osteosarcoma, COS-33, mTOR, osteogenic differentiation, TP53

## Abstract

Outcomes have not improved for metastatic osteosarcoma for several decades. In part, this failure to develop better therapies stems from a lack of understanding of osteosarcoma biology, given the rarity of the disease and the high genetic heterogeneity at the time of diagnosis. We report here the successful establishment of a new human osteosarcoma cell line, COS-33, from a patient-derived xenograft and demonstrate retention of the biological features of the original tumor. We found high mTOR signaling activity in the cultured cells, which were sensitive to a small molecule inhibitor, rapamycin, a suppressor of the mTOR pathway. Suppressed mTOR signaling after treatment with rapamycin was confirmed by decreased phosphorylation of the S6 ribosomal protein. Increasing concentrations of rapamycin progressively inhibited cell proliferation *in vitro*. We observed significant inhibitory effects of the drug on cell migration, invasion, and colony formation in the cultured cells. Furthermore, we found that only a strong osteogenic inducer, bone morphogenetic protein-2, promoted the cells to differentiate into mature mineralizing osteoblasts, indicating that the COS-33 cell line may have impaired osteoblast differentiation. Grafted COS-33 cells exhibited features typical of osteosarcoma, such as production of osteoid and tumorigenicity *in vivo*. In addition, we revealed that the COS-33 cell line retained a complex karyotype, a homozygous deletion of the *TP53* gene, and typical histological features from its original tumor. Our novel cellular model may provide a valuable platform for studying the etiology and molecular pathogenesis of osteosarcoma as well as for testing novel drugs for future genome-informed targeted therapy.

## INTRODUCTION

Osteosarcoma (OS) is the most common primary bone cancer in adolescents and in the elderly. Each year, approximately 800 to 900 new cases are diagnosed in the United States [1]. Current multimodal therapy consisting of surgery combined with drug treatment using chemotherapy agents (cisplatin, doxorubicin, ifosfamide, and high-dose methotrexate) achieves a five-year survival rate of approximately 60–70% for localized OS, while the rate for patients with metastases is less than 30% [2]. Recent genomic studies with patient tumor samples and models of human OS have improved our understanding of the disease etiology and have also provided a road map for drug development [3–6]. However, the clinical and survival outcomes for patients with OS have not improved over the last four decades. This is in part due to the rarity of the disease and the high genetic heterogeneity at diagnosis, which together challenge us to better understand OS biology in order to make discoveries that can improve clinical care [7]. One way to overcome the difficulty in assessing OS patient samples is to establish new cell lines and to thoroughly characterize the existing models.

OS cell lines, which are excellent experimental systems, are important for basic and preclinical studies as they allow the investigation of general cell biology and drug discovery. Since the establishment of the first human OS cell line, named U-2 OS (or 2T), in 1967, many human OS cell lines, such as SJSA-1 (or OSA), MG-63, and Saos-2, have been successfully established and characterized [8–14]. Among them, Saos-2 contains homozygous deletion mutations of *TP53*, whereas the others express the wild-type gene [15]. Nonetheless, it is still unknown how *TP53* mutation status affects therapeutic strategies and overall patient survival [4, 5]. Due to the rarity of the disease, establishing novel OS cancer cell lines representative of the extensive heterogeneity of these tumors will likely provide additional insights and serve as valuable platforms for developing effective therapies.

Previous studies have demonstrated that many features of OS such as cytogenetic abnormalities, histologic integrity and subtypes, and mRNA expression profiles are retained in OS cell lines and/or patient-derived xenografts (PDXs) [12, 16]. This suggests that they accurately reflect genetic and biologic characteristics of the primary tumors from which they are derived. Therefore, they are useful alternatives to experimental animal tumor models. Over the past 30 years, numerous groups have used models of PDXs for basic and preclinical studies, including the Pediatric Preclinical Testing Consortium (PPTC), previously known as the Pediatric Preclinical Testing Program [9, 16–19]. One of the lines was named OS-33 (or HxOS-33), but it has seldom been grown and studied in culture [4, 20–24]. In this study, we report the successful establishment of a novel human OS cell line derived from OS-33, herein designated COS-33, and demonstrate retention of the biological features and drug sensitivity of the original PDX tumors.

## RESULTS

### A newly established COS-33 cell line shows high mTOR signaling activity and is sensitive to rapamycin

**Figure 1:**
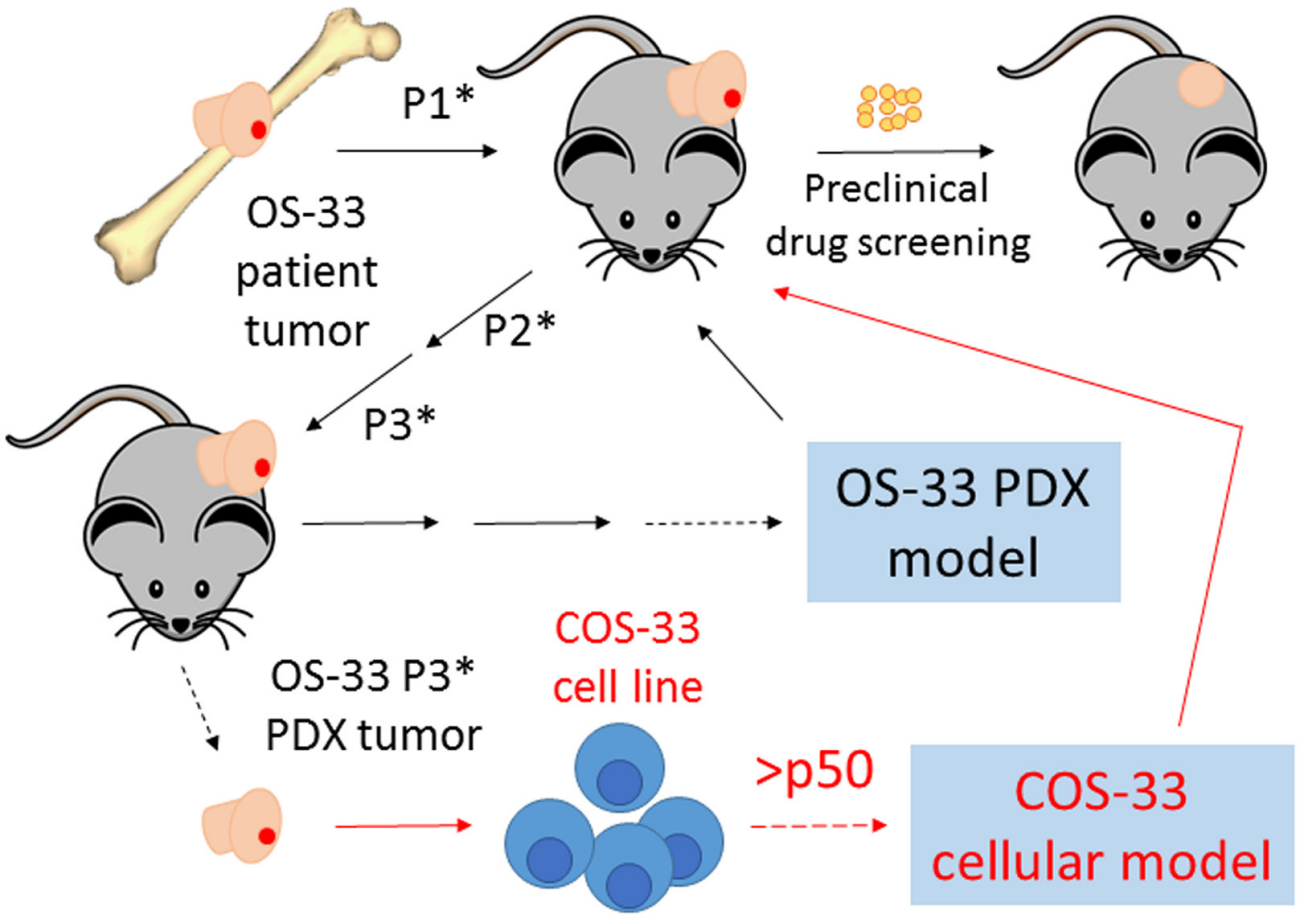
Schematic diagram summarizing how our novel cell line, COS-33, was established. This figure includes an explanation of our previously described work establishing the patient-derived xenograft (PDX) mouse model [16]. The cartoon on the top left side, with the black arrow lines, shows that immunodeficient mice were subcutaneously implanted with the primary osteosarcomas obtained from a seven-year-old girl after definitive surgery, but prior to chemotherapy. Successful grafted human tumors propagated in mice in passage 1 (P1^*^), passage 2 (P2^*^), passage 3 (P3^*^), and later passages (black line). A PDX tumor called OS-33 was chosen to be an *in vivo* mouse tumor model in the Pediatric Preclinical Testing Consortium and has been used to test numerous anticancer agents, including rapamycin [19]. In this study, we established a novel cell line, COS-33, from a P3^*^ tumor of OS-33. The cartoon on the bottom right side with red arrow lines shows that the primary cells have been propagated in *in vitro* cell culture more than 50 passages so far. The COS-33 line can be used as a cellular model to study OS cell biology and to screen for cancer drugs.

**Figure 2:**
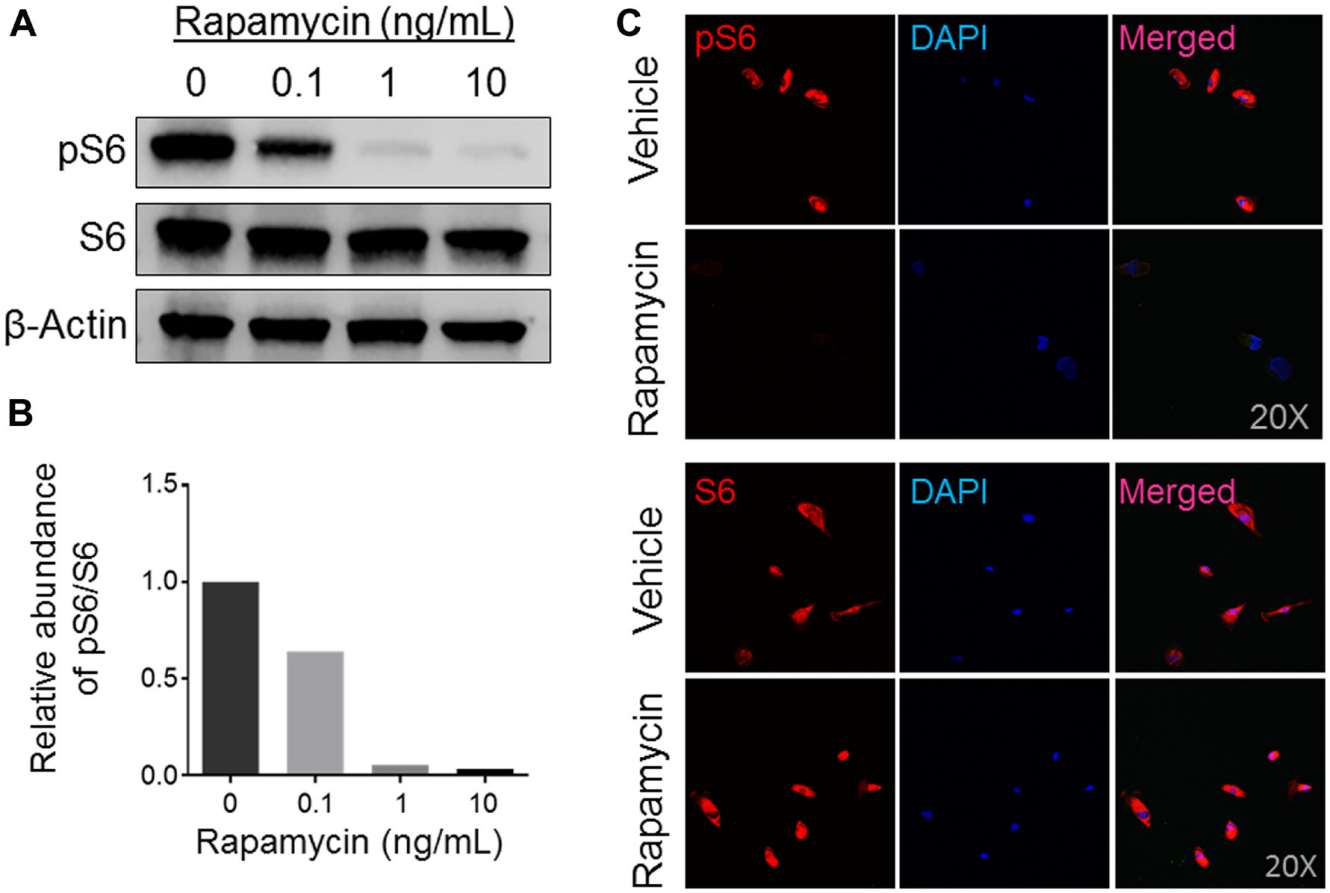
Expression analysis of mTOR activity in rapamycin-treated human osteosarcoma COS-33 cells. (**A**) Western blotting analysis of total S6 and phosphorylated S6 (pS6) protein levels to determine activity of the mTOR pathway in human COS-33 cells treated with varying amounts of rapamycin (0, 0.1, 1, 10 ng/mL). (**B**) Quantification of (A). (**C**) Immunofluorescence staining of cells treated with rapamycin (1 ng/mL) and then probed with both pS6 and total-S6 antibodies, respectively, shows a decrease in mTOR activity. The images were taken under 20× objective magnification.

Recent next-generation sequencing data analyses of OS in human and mice from our laboratory and of others’ suggest that mTOR pathway kinases possess mutations and/or high expression levels and are potential targets for small molecule inhibitors [3, 6, 25, 26]. We opted to establish and characterize a cell line derived from a previous established PDX model in this study because of its good response (maintained complete regression) to rapamycin monotherapy in the initial testing (stage 1) conducted by the PPTC (Figure 1) [19]. Rapamycin (or Rapa), an antibiotic macrocyclic lactone, is a highly specific inhibitor of mTOR, a serine/threonine kinase that leads to phosphorylation of the S6 ribosomal protein (from S6 to pS6) during its cap-dependent translation. To examine whether our newly generated COS-33 cell line retains high mTOR signaling activity and is sensitive to rapamycin, we performed Western blotting and immunostaining analysis using antibodies against S6 and pS6, respectively. The pS6 level decreased as the drug concentrations increased, signifying that the mTOR pathway inhibition is concentration-dependent, with a concentration of 1 ng/mL sufficient for significant inhibition (Figure 2A and 2B). Immunofluorescence staining with this concentration was also performed to detect whether this compound inhibited mTOR activity in the COS-33 cell line. Our immunostaining results support the Western blotting data, as there appears to be significantly lower pS6 in the treated cells compared to the vehicle control (Figure 2C).

### Rapamycin treatment inhibits COS-33 cell proliferation and colony formation

**Figure 3:**
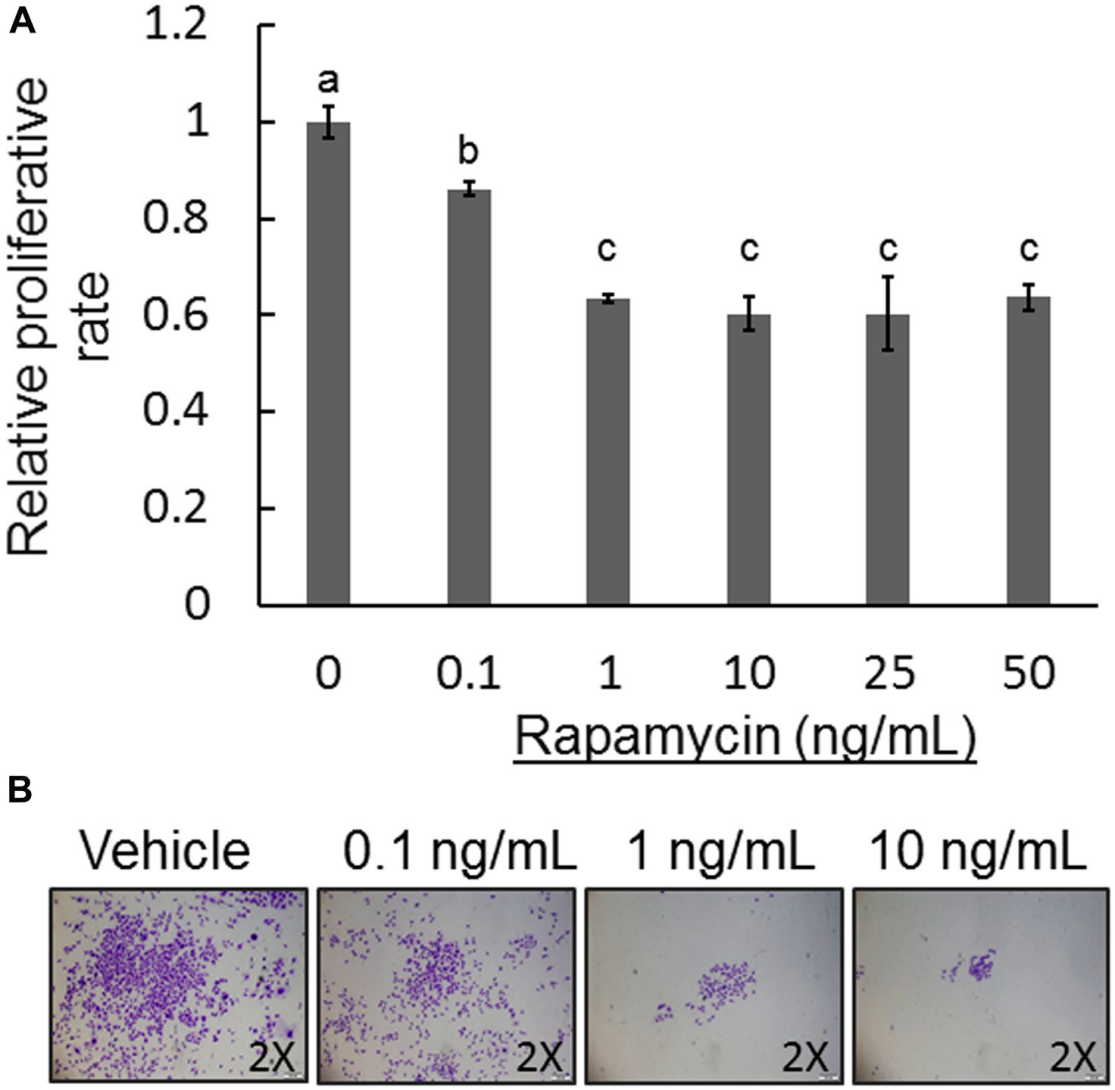
Effect of rapamycin on COS-33 cell proliferation and colony formation. (**A**) Five different concentrations of rapamycin (0.1, 1, 10, 25, and 50 ng/mL) were used to examine the inhibitory effect on the COS-33 cell line. Unique letters above bars represent significant differences between the groups, and means with the same letter are not significantly different from each other (ANOVA with Tuckey HSD test, *p* < 0.05). 1 ng/mL rapamycin was adequate to significantly inhibit proliferation. (**B**) A colony formation assay was performed on COS-33 cells treated with different concentrations (0.1, 1, 10, and 20 ng/mL) for 14 days. Colonies were then fixed and stained with 0.5% Crystal Violet and images were taken with an inverted microscope under 2× objective magnification.

The activated mTOR pathway has key roles in cell growth and survival in human OS [19]. To gain a better understanding of whether COS-33 cells in culture retain their cellular functions in a manner similar to that observed *in vivo*, we performed cell proliferation, migration, invasion, colony formation, and osteoblast differentiation assays with or without treatment of rapamycin. We first determined the effective concentration of rapamycin on the cell line by performing a proliferation assay with five concentrations (0.1, 1, 10, 20, and 50 ng/mL), which were similar to those used in *in vitro* testing of rapamycin for a panel of 23 cell lines [19]. The 0.1 ng/mL concentration showed significant inhibition (*p* < 0.05), although it was not as effective as the higher concentrations, at 72 h (Figure 3A). The 1 ng/mL concentration significantly suppressed cell proliferation, with the three higher concentrations seemingly varying little at 24, 48, and 72 h (Figure 3 and data not shown). Thus, 1 ng/mL was used in our cellular functional experiments thereafter.

To study the role of mTOR signaling in COS-33 cell development, we performed a colony formation assay using rapamycin-treated COS-33 cells. Crystal Violet staining clearly showed that treatment with rapamycin inhibited clonogenicity of COS-33 cells in a concentration-dependent manner with increasing rapamycin concentrations from 0.1 to 10 ng/mL (Figure 3B). Because clonogenicity is a sensitive indicator of undifferentiated cancer stem cells (CSCs), this result implies that mTOR signaling may be required for CSC self-renewal and anchorage-independent growth in order to maintain CSC populations in tumor tissues.

### mTOR signaling activity is involved in cell migration and invasion

**Figure 4:**
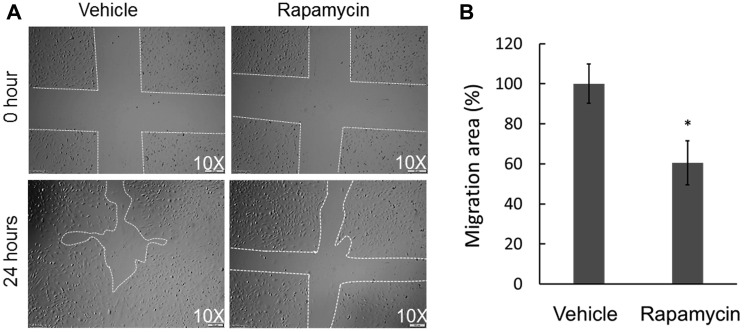
Effect of rapamycin on the *in vitro* wound healing ability of COS-33 cells. (**A**) Representative pictures of wound repair 24 h after the mechanical scratch (under 10× objective magnification; left: vehicle, right: treated with 1 ng/mL). The white lines indicate the edges of the wounded area. (**B**) Quantitative analysis of the wound repair 24 h after the scratch. Wound healing area of vehicle-treated cells is defined as 100% versus areas of drug-treated cells (^*^ *p* < 0.05).

**Figure 5:**
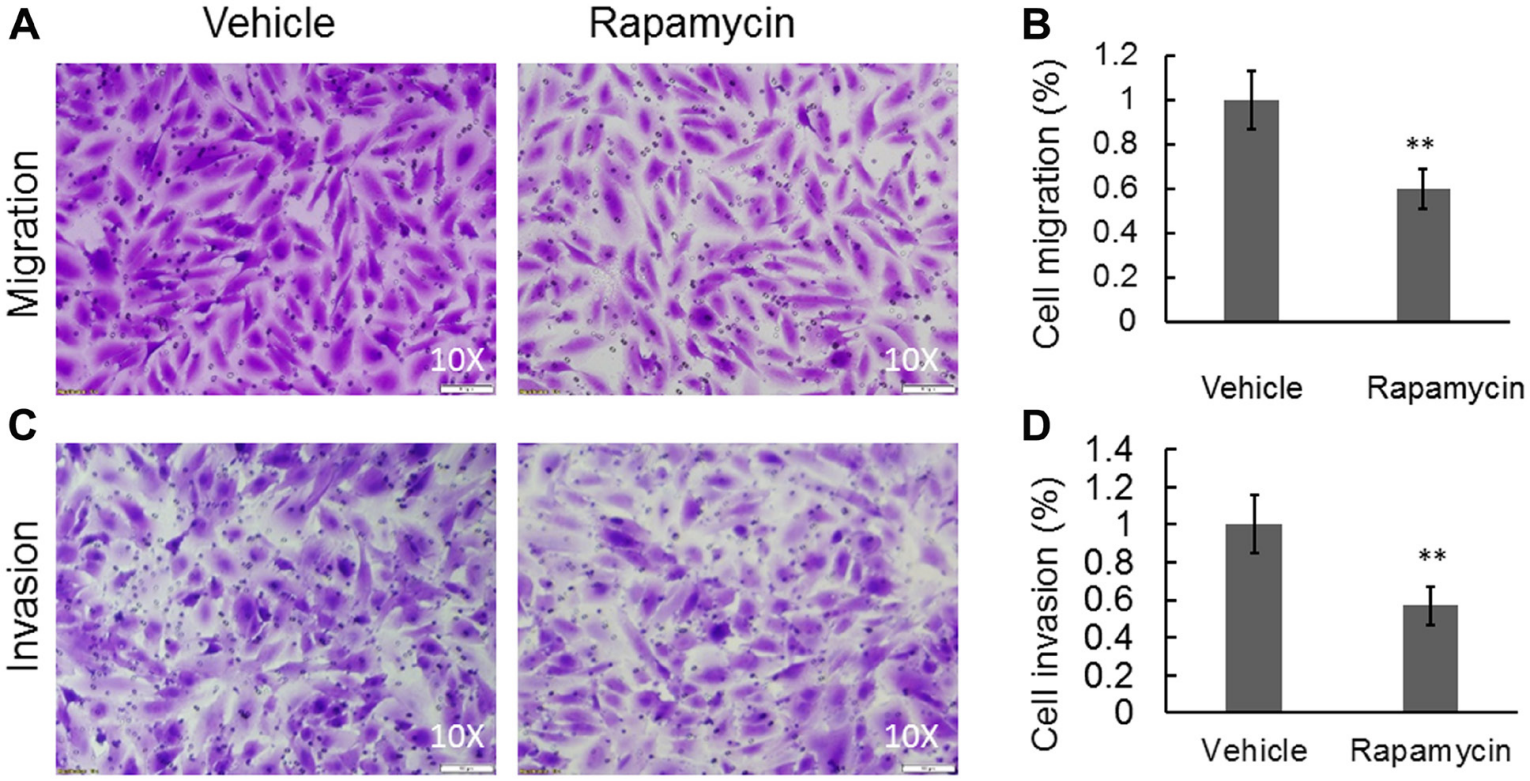
Rapamycin was able to significantly inhibit cell migration and invasion in a Boyden chamber-based system. (**A**) Cell migration and (**C**) cell invasion were detected using a Boyden chamber-based system following treatments with either vehicle or 1 ng/mL rapamycin. (**B**) Quantification of (A). (**D**) Quantification of (C). Data are expressed as the means ± standard deviation; *n* = 3. ^**^ *p* < 0.01 compared with control vehicle group.

OS metastasis formation requires a precisely orchestrated regulation of multiple cellular processes that involve cell migration and invasion. To examine the inhibitory effects of rapamycin on COS-33 cells’ ability to migrate, we first performed a wound healing assay. After 24 h, the cells treated with rapamycin showed a significantly decreased ability to close the wound, in comparison to those treated with vehicle (Figure 4A, 4B). To further examine this observation, we employed a Boyden chamber-based cell migration system (or filter membrane migration assay) to examine cell migration behavior following rapamycin treatment [27]. Quantification of the cells that migrated through the insert membrane showed an approximate 40% reduction after rapamycin treatment as compared to the control (Figure 5A, 5B). To further investigate the invasion ability of the cells, we added a thin layer of Matrigel to the top insert of the chamber, which mimics the extracellular matrix of the tumor cell environment *in vivo* [28]. The number of treated cell significantly decreased compared to the vehicle control (Figure 5C, 5D), indicating an inhibitory effect of rapamycin on COS-33 cell invasion. Our data showing the inhibitory effects of rapamycin on cell migration *in vitro* are consistent with the previous testing of the *in vivo* antimetastasis efficacy of rapamycin in OS [23].

### BMP2 promotes COS-33 cells to differentiate into mature mineralizing osteoblasts

**Figure 6:**
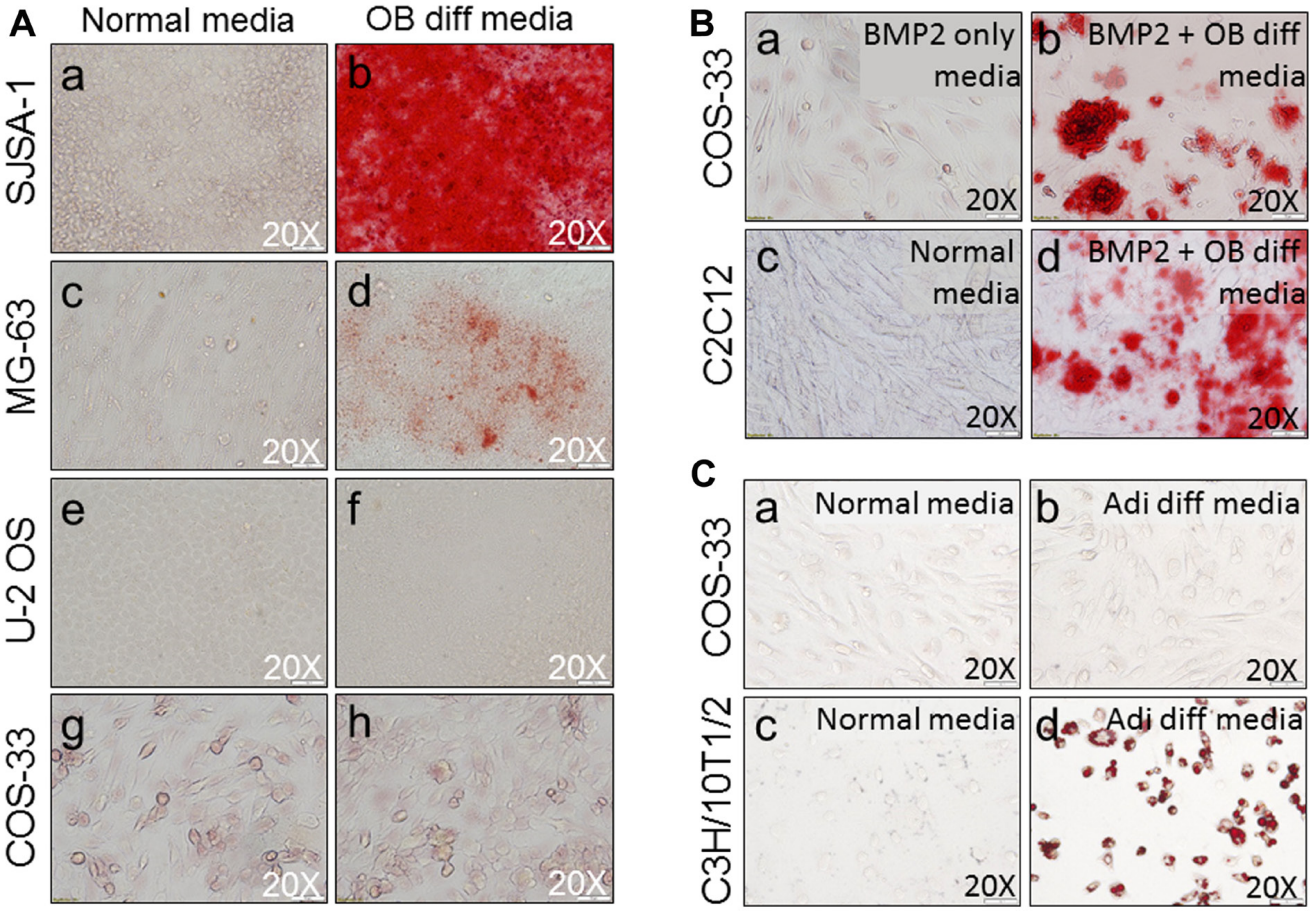
Functional characterization of the cells by osteogenic and adipocyte differentiation assays. (**A**) Osteogenic differentiation (OB diff) assay shows similarities and differences between COS-33 cells and other established cell lines. Representative pictures of cell cultures under normal conditions (a, c, e, g) & osteoblastic differentiation conditions (b, d, f, h) show the morphology of cells after Alizarin Red S staining for osteoblastic mineralization in SJSA-1 (a-b), MG-63 (c-d), and U-2 OS (e-f), and COS-33 (g-h). (**B**) addition of BMP2 promotes osteogenic differentiation of COS-33 and C2C12 cells. Cells were cultured for 14 days with or without a strong osteogenic inducer (BMP2) and stained with Alizarin Red and photographed. (a) COS-33 treated with 100 ng/mL BMP2 only; (b) COS-33 under osteoblastic conditions with 100 ng/mL BMP2; (c) C2C12 under normal conditions; and (d) C2C12 under osteoblastic conditions with 100 ng/mL BMP2. (**C**) The adipocyte differentiation (Adi diff) assay shows differences between COS-33 cells and an established cell line, C3H/10T1/2. Representative pictures of cell cultures under normal conditions (a, c) & adipocyte differentiation conditions (b, d) show the morphology of cells after Oil Red O staining for lipid droplets in COS-33 (a-b) and C3H/10T1/2 (c-d). All images were taken under 20× objective magnification.

To examine the osteogenic differentiation capacity of COS-33 cancer cells, we performed an osteoblast (OB) differentiation assay. First, we compared OB differentiation capability to other human OS cell lines (i. e. SJSA-1, MG-63, and U-2 OS). OB differentiation was assessed by the detection of mineralized bone matrix by Alizarin Red staining. SJSA-1 showed a robust differentiation capability when treated with the differentiation medium (Figure 6Aa-b), while MG-63 appeared to have a limited capability (Figure 6Ac-d) and U-2 OS had none (Figure 6Ae-f). BMP2 is known to have a strong osteogenic capacity both *in vitro* and *in vivo*, and it promoted osteogenic differentiation of C2C12 cells, a myoblast precursor cell line that has the ability to differentiate into bone cells in response to BMPs, in our previous studies [29, 30]. When treated with the OB differentiation medium alone, or BMP2 only, COS-33 cells did not differentiate into mature osteoblasts (Figure 6Ag-h and 6Ba), similar to U-2 OS. With the addition of BMP2 to the OB differentiation medium, COS-33 cells showed a capability to differentiate into mineralizing osteoblasts, which produce bone matrix and calcium (Figure 6Bb), similar to C2C12 (Figure 6Bc-d). In addition, we performed an adipocyte differentiation assay and found that COS-33 cells have little adipocyte differentiation capability (Figure 6Ca-b), which was in contrast to C3H/10T1/2, a typical pluripotent mesenchymal precursor cell line that has the ability to differentiate into fat cells under adipocyte differentiation conditions (Figure 6Bc-d). This suggests that the cultured COS-33 cells may possess an active osteoblastic cell differentiation program, although it is impaired.

### The COS-33 cell line retains similar karyotype, TP53 mutation status, and histological features, including presence of osteoid matrix, to the parental PDX tumor

**Figure 7:**
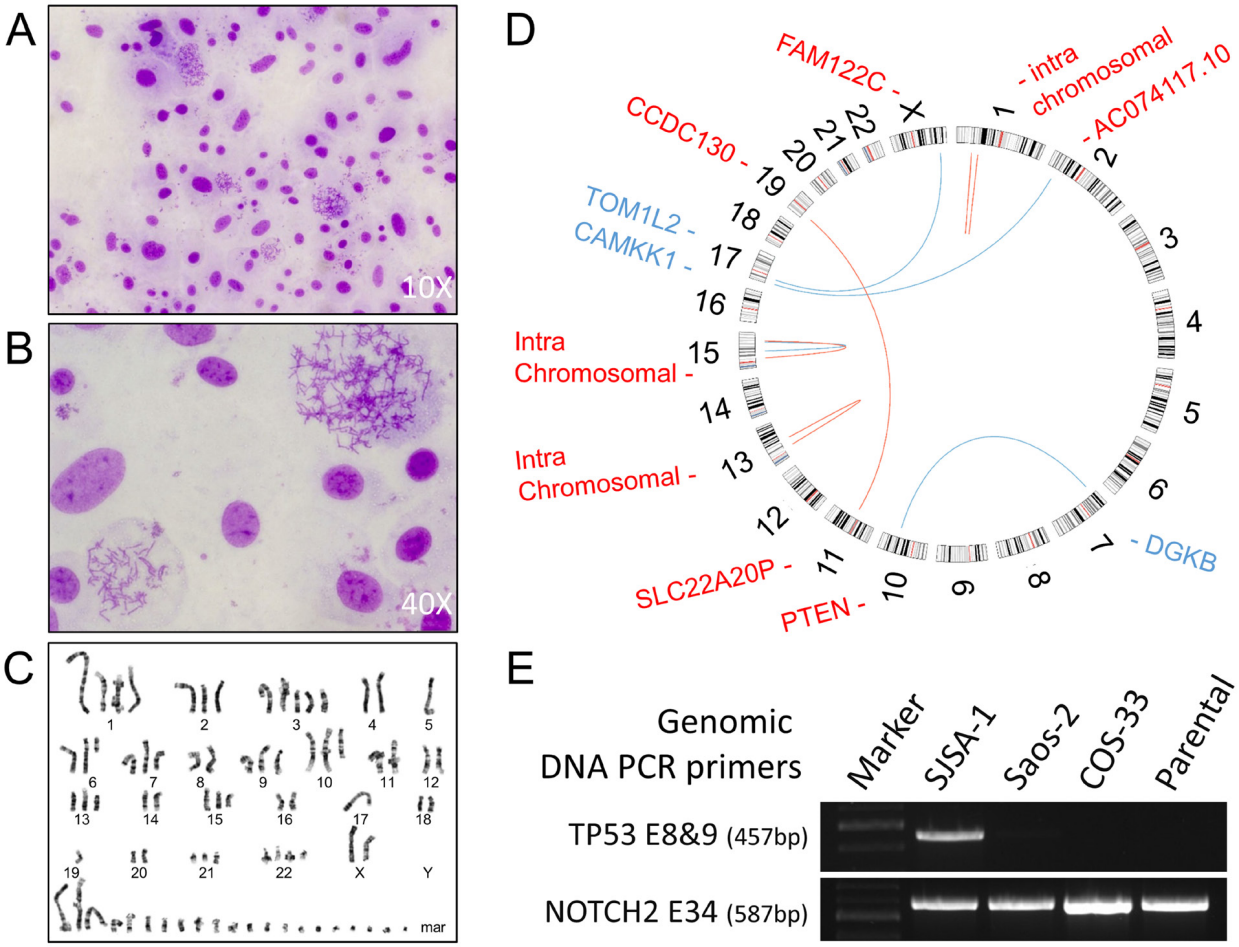
Conventional cytogenetics analysis and genomic analysis. (**A**, **B**) Images taken from representative Giemsa-stained metaphase spreads used for karyotyping at 10× (A) and 40× (B); (**C**) A representative G-banded karyotype of a COS-33 cell. COS-33 cells showed extreme abnormalities typically found in OS samples. (**D**) Circos plot for PDX OS-33 tumor. The red lines are +/+ strand fusion, whereas blue ones are +/− strand fusions. Genes highlighted in blue color are located in the minus strand, whereas the ones highlighted in red are in the plus strand. Four pairs of genes with intrachromosomal translocations are not listed here but can be found in the Supplementary Table 1. (**E**) Images of agarose gel electrophoresis of PCR fragments for amplification of *TP53* exon 8 & 9 and NOTCH2 exon 34 in the parental PDX and COS-33 cell line, using a *TP53* wild-type control (SJSA-1) and *TP53* deletion mutation control (Saos-2).

To understand the degree of genomic instability of COS-33 cells, we performed conventional cytogenetic analysis and *TP53* mutation screening (Figure 7A, 7B). A representative karyotype is shown in Figure 7C. We analyzed 20 COS-33 cells, which exhibited hyper-triploid clones with several rearranged chromosomes. Among the 20 analyzed metaphases, 13 cells showed a range of 71−83 chromosomes, and the remaining 7 cells showed a range of 143–155 chromosomes. The precise identification of the structural abnormalities was not possible due to the complexity of the rearranged chromosomes. Our data are consistent with a previous study that used cells directly from the parental OS-33 PDX tumor [16]. Moreover, this complex karyotype was corroborated by gene fusions identified in the PDX model using deposited whole exome sequence data (Figure 7D and Supplementary Table 1). All fusions involved a chromosome with either gain or loss, suggesting a close association between chromosomal instability and structural alterations.

To examine the *TP53* gene mutation status in COS-33 cells, we performed Sanger sequencing of genomic DNA PCR amplicons targeting *TP53* exons 8 to 9, which code a portion of the DNA-binding and tetramerization domains of the TP53 protein. A single 457-bp PCR product was amplified using a forward primer within intron 7 and a reverse primer within intron 9. This *TP53* amplicon was not detected in the Saos-2 and COS-33 cell lines or the parental PDX tumor but was present in the SJSA-1 line (Figure 7E). A NOTCH2 PCR amplicon (587 bp), which was amplified using a pair of primers within exon 34, was present in all three cell lines. The results from Saos-2 and SJSA-1 are consistent with the previous report that Saos-2 line, but not SJSA-1 (*TP53*^wt^), harbored a deletion mutation of exons 4−8 [15]. Our data suggests that the novel COS-33 line may harbor a homozygous deletion affecting exons 8−9 of *TP53* gene.

**Figure 8:**
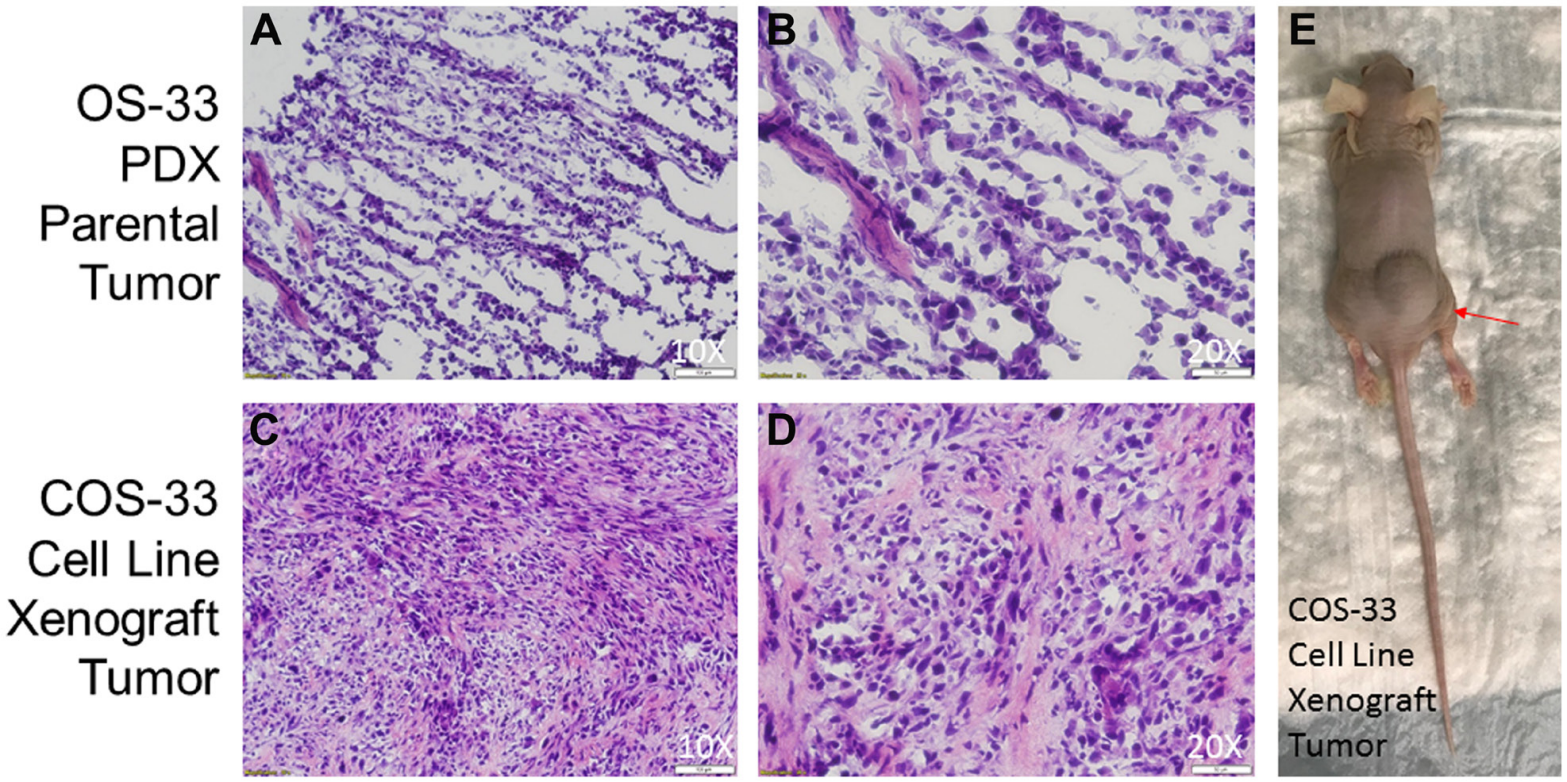
Photographs of histological sections of COS-33 tumors and the xenograft mouse. (**A**–**B**) Representative images of hematoxylin-and-eosin (H&E) stained section of the parental PDX tumor from which the COS-33 cells were derived. (**C**–**E**) Representative images of H&E stained section of the COS-33 cell-line-grafted tumor and a tumor-bearing nude mouse (E). The objective magnification 10× (A and C) and 20× (B and D).

To understand their tumorigenic potential, COS-33 cells were grafted into immunocompromised mice. The tumors were harvested and analyzed with H&E staining alone, as the parental PDX tumor from which the cell line was derived (Figure 8). The resulting tumors from COS-33 cells closely resembled the parental PDX OS. Development of the osteoid matrix was clearly visible in both types of samples (Figure 8A–8D), as were similarities in the shape and size of the cells between the parental tumor (Figure 8A, 8B) and the COS-33 cells-xenograft tumor (Figure 8C, 8D). However, tumors from both the COS-33 and the PDX were poorly differentiated, since only small regions of the tumor produced osteoid (bone matrix prior to calcification). Together, these data suggest that the COS-33 cell line maintains genomic, histological, and cytological features, including presence of osteoid matrix, that are similar to the parental tumor, as well as a complex karyotype and *TP53* gene mutation.

## DISCUSSION

Our present study describes the establishment and characterization of a novel OS cell line. We showed that mTOR inhibitor rapamycin inhibits COS-33 cell proliferation, migration, invasion, and clonogenicity. We also demonstrated that the cell line retains its osteoblastic cells of origin, karyotype, *TP53* mutation status, and histological features in comparison to the parental PDX tumor from which it originates. Our novel cellular model may provide a valuable platform for studying OS malignancies, general cell biology, and drug discovery.

In the past 30 years, PDXs of human OS have served as a valuable tool for basic and clinically applied research, especially when one ponders the difficulty of establishing OS cell lines *in vitro*. However, *in vitro* permanent cell lines provide an unlimited, self-replicating source of cells that can be widely distributed to facilitate comparative studies, especially considering the rarity of OS. Most human OS cell lines were derived from fresh tumors from patients, including SJSA-1, MG-63, U-2 OS and Saos-2 [8–14]. In the present study, we successfully established the COS-33 cell line, which was derived from an osteoblastic PDX tumor line in its third passage (Figure 1). The COS-33 cells can be used to test new drugs with a combination of chemotherapeutic drugs. Because the original tumor tissues came from an untreated female patient [16], the COS-33 cell model may represent naïve disease and may enable the identification of novel and effective agents for front-line therapy. The COS-33 cell line was subcultured for more than 50 passages without obvious changes in morphology or proliferative potential after cryopreservation and resuscitation. The cytological features of COS-33 cells in the grafted tumor are similar to those in the parental PDX tumor (Figure 8), and previous studies demonstrated that the PDX tumors accurately reflect the genetic and biologic characteristics of the tumor of origin [4, 16]. Thus, COS-33 cells may constitute an excellent *in vitro* and *in vivo* model representative of the original tumor.

A major challenge in treating OS is that there are no secondary lines of agents available for patients who are unsuitable for intensive chemotherapy and surgical regimens. Treatment modalities such as blockade of signaling pathways, including mTOR, are arising as a promising approach in the treatment of OS [3, 19]. mTOR is an intracellular serine/threonine kinase involved in the PI3K/AKT pathway, that indirectly regulates ribosomal translation of mRNA into proteins necessary for cell growth, cell cycle progression, and cell metabolism, making it an excellent target for cancer cells to hijack. Because of its demonstrable response to mTOR-targeted rapamycin monotherapy, the PDX OS-33 model has been frequently used in many studies to test new mTOR inhibitors (e. g. temsirolimus), and the activity of combinations of rapamycin with other anticancer agents such as cisplatin, bevacizumab, eribulin (against the tubulin binding agent), and R1507 (a fully human monoclonal antibody targeting IGF-1R) [4, 20–24]. In addition, it was used to test new drugs inhibiting other signaling pathways such as STAT3 [17, 22].

Our newly established COS-33 line can provide a complementary or new tool to screen new drugs and to perform a relatively rapid *in vitro* test on efficacy of combination therapy with rapamycin and other therapeutic agents. For example, we and others have shown that Wnt-targeted monotherapies (PRI-724 or tegavivint) can reduce OS growth *in vitro* or *in vivo* [31, 32]. A combination therapy with inhibitors of both mTOR and Wnt pathways warrants further study, as together they play a key role in cancer metastasis formation and chemoresistance.

Based on cytological and bone matrix features, human OS can be classified into at least three subtypes: osteoblastic (50%), chondroblastic, and fibroblastic [2, 3]. The original tumor that generated the COS-33 cells belongs to the osteoblastic subtype [16]. However, it is difficult to determine from which stage the tumor-initiating cell of COS-33 arose, because any cell present during mesenchymal stem cell/osteoblast differentiation can serve as a candidate for the cell of origin. On the other hand, we found that the cultured cells can only undergo osteogenic differentiation after the addition of a stronger inducer of osteogenesis, BMP2 (Figure 6), suggesting that the program of osteoblast differentiation has been at least partially blocked by gene mutations accumulated in OS-33 cells. Interestingly, the impaired *in vitro* cell differentiation capacity of OS-33 may resemble the low differentiation status of the original bone tumor, which was manifested as a highly cellular tumor with rare areas of calcified osteoid [16]. Mechanistically, the blockade of osteogenic differentiation is likely caused by loss of tumor suppressors *TP53* and *RB1*, which have an ability to strongly influence osteoblastic differentiation in normal bone cells and OS cells [33–36].

*TP53* and *RB1* mutations serving as initiating cancer drivers have been identified in inherited familial Li-Fraumeni syndrome and hereditary retinoblastoma with a predisposition to OS [3]. Next-generation sequencing studies have found that recurrent somatic alterations of these two genes are present in more than half of the bone tumors [25, 37]. The presence of p53 mutations in human OS correlates with high levels of genomic instability [38]. Consistent with this, our COS-33 cell line showed abnormal complex karyotypes, including increases in chromosome number as well as chromosomal breaks and translocations (Figure 7C). Overholtzer *et al*. originally reported a mutation in exon 8 of *TP53* in the parental tumor of COS-33 cells [38]. To verify whether our cell line retains this mutation, we examined this region but found that the complete or part of the region was not detected, suggesting that there is a homozygous deletion mutation of *TP53* in COS-33 cell line (Figure 7E). A more recent study using the whole exome sequence approach described by Rokita *et al*. supports our conclusion [4]. Moreover, an RB1 gene mutation has been found in the OS-33 PDX tumor in this study. However, according to the “three drivers” model we recently proposed, an additional cancer driver, in addition to TP53 and RB1, may be required for full-blown OS at the time of diagnosis; one or more oncogenic drivers within the cohort of recurrent somatic copy-number alterations or fusion gene products may fulfill this role [3, 39]. For example, a fusion transcript and protein product from the PTEN-DGKB gene fusion may be a causative driver and warrants further investigation. Targeting those oncogenic drivers using a genome-matched approach proved in a recent study by the Sweet-Cordero’s group may provide a road map for treatment of OS [5]. Future identification of the oncogenic drivers in our COS-33 cell line, and testing combination therapy of rapamycin together with agents inhibiting the identified drivers, should facilitate further understanding of the principle of genome-informed targeted therapy for OS.

In summary, we established a novel permanent human cancer cell line, COS-33, originating from a female patient who was diagnosed with osteoblastic OS. The cell line retains the characteristics of the original tumor, including histopathology, cytogenetic complexity, osteoblastic activity, and drug sensitivity. The COS-33 cell line can serve as a new model for investigating the etiology and molecular pathogenesis of OS and for testing novel drugs for treatment.

## MATERIALS AND METHODS

### Source of tumors, establishment of the tumor cell line, and transplant assays

In the present study, we have established the new COS-33 cell line. It originates from a third-passage tumor tissue of a PDX model line, which was maintained by the PPTC (San Antonio TX). The parental PDX model (called OS-33) was created by direct transplantation of a fraction of a patient primary tumor (untreated) into an immunodeficient mouse, without any previous *in vitro* culture or clonal selection, at St. Jude Children’s Research Hospital, where it was first reported in 1989 [9, 16]. The patient was a seven-year old female of European descent. The tumor originated in her humerus and presented with an osteoblastic histopathological appearance.

To establish the COS-33 cell line, the parental xenograft tumor pieces were digested using a mix of serum-free αMEM medium (Hyclone, SH30265FS), Collagenase P (Sigma, 11213857001), Trypsin-EDTA (Gibco, 25-200-056), and Penicillin-streptomycin (Hyclone, SV30010) (Figure 1). The digested tumor cells were then seeded into a 100-mm tissue culture dish (Fisher Scientific, 430167). Cells were initially cultured in the growth medium containing 20% Fetal Bovine Serum (FBS) (Fisher Scientific, ES009B) and 1% Penicillin-streptomycin at 37°C in a humidified atmosphere containing 5% CO_2_. After 48 h, the cells were switched to a growth medium containing 10%FBS. The cells were grown to confluence and then subsequently passaged using Trypsin-EDTA. All assays were performed using cells after 30 passages, unless otherwise stated.

For transplant assays, COS-33 cells were grown to confluence in 150-mm plates (Corning, 430599) and then rinsed with phosphate buffered saline (PBS) (Fisher Scientific, MT21040CV). A volume of 5 mL of serum-free medium was added to the plate, and the cells were scraped off using a cell scraper (Fisher Scientific, 02-683-197). The cells were then pelleted in a 15-mL centrifuge tube (Fisher Scientific, 14-959-70C) at 1000xg for 2 min, and the supernatant was removed before resuspension of the pellet in 300 μL of serum-free medium per plate scraped. The cells were then subcutaneously (s. c.) injected on the flank, near the hind limb of nude mice. Tumors were then allowed to grow over the next few months and were harvested when their size reached 2 cm in diameter. Samples were collected from the tumors for hematoxylin and eosin staining (H&E), and other samples were harvested for future transplant assays and for the derivation of a cell line. These studies were conducted in accordance with the National Institutes of Health Guide for the Care and Use of Laboratory Animals and approved by the Sanford Research Institutional Animal Care and Use Committee.

### Cell culture and treatment with mTOR inhibitor

Cell lines Saos-2 (HTB-85), SJSA-1 (CRL-2098), U-2 OS (HTB-96), MG-63 (CRL-1427), C3H/10T1/2 Clone 8 (CCL-226), and C2C12 (CRL-1772) were all purchased from the American Type Culture Collection and maintained in growth medium containing 10% FBS (Fisher Scientific, ES009B) and 1% Penicillin-streptomycin (Hyclone, SV30010) at 37°C under a humidified atmosphere containing 5% CO_2_. Unless stated otherwise, cells in 100-mm tissue culture dishes (Fisher Scientific, 430167) were treated with 1 ng/mL rapamycin (AdooQ Bioscience, A10782) for 24 h. The corresponding amount of dimethyl sulfoxide (DMSO) or ethanol was used as a vehicle control.

### Western blot and immunostaining analysis

Cells were seeded into a 100-mm dish and treated with 0, 0.1, 1, and 10 ng/mL rapamycin or vehicle, respectively, for 24 h. Western blotting was performed according to the modified procedure previously described [31]. Cells were briefly lysed with a 1× Laemmli Sample Buffer solution (Biorad, 1610737). Lysates were separated with 4–20% Mini-PROTEAN^®^ TGX™ Precast Protein Gels (Biorad, 4561094) and transferred onto a PVDF membrane (BioRad, 1704272) using a semi-dry Bio-Rad Trans-blot Turbo apparatus. The membranes were first probed with rabbit anti-pS6 antibody (Cell Signaling Technology, 5364S), rabbit anti-S6 antibody (Cell Signaling Technology, 2217S), and anti-β-actin (Santa Cruz Biotechnology, sc-47778) primary antibodies. After incubating the membranes with goat anti-mouse and goat anti-rabbit Li-Cor IRDye 800 (green) and 680 (red) secondary antibodies (1:10,000; Li-Cor, Lincoln, NE, USA) in blocking buffer for 1 h at room temperature, protein bands were visualized on an Odyssey imaging system (Li-Cor, Lincoln, NE, USA) and quantified with the optical density (OD) function of Image J software (NIH, Bethesda, MD, USA). Immunostaining analyses were performed as described before [6]. Cells were seeded into a 4-well chamber slide with removable wells (Fisher Scientific, 12-565-7) at a density of 3.0 × 10^5^ cells, with some of the wells receiving 1 ng/mL rapamycin for 24 h. The following day the cells were rinsed and fixed with formalin (Cardinal Health, C4320-101). The wells were then probed with the rabbit anti-S6 and rabbit anti-pS6 used in Western blotting. The wells were rinsed again and blocked with normal goat serum (ThermoFisher, 31872). The secondary antibody was then added, and the slides were put into a light-blocking box, with some water added to prevent evaporation, at 4°C overnight. The following day, anti-fade mounting medium with DAPI (Vector Labs, H-1200) was applied after rinsing the secondary antibody off, and the slide was imaged using a Nikon A1 Confocal microscope.

### CCK-8 cell proliferation assay and colony formation assay

The cell proliferation CCK-8 assays (Dojindo Molecular Technologies, CK04-11, Kumamoto, Japan) were performed according to the manufacturer’s instructions. Cells were seeded into a 96-well plate (Fisher Scientific, N8010560) at a density of 6,250 cells per well as five replicates, with medium containing no cells serving as a control. Cells were treated with rapamycin (or vehicle) at 0, 0.1, 1, 10, 25, 50 ng/mL for 72 h, with media changes at 24 and 48 h. Then, 10 μL of the CCK-8 reagent was added to each well, including wells with just medium as negative controls, once the cells reached 72 h. The plates were put back into the incubator for 1 h. The plates were then read for OD using the Cytation3 at 450 nm. All experiments were performed with at least three independent replicates. For colony formation assay, cells were seeded into a 6-well plate at a density of 1,000 cells/mL. The cells were treated with rapamycin (10, 1, 0.1 ng/mL) or vehicle and put back into the incubator and cultured for two weeks, or until visible clonal colonies formed. The colonies were washed with PBS, fixed with 10% formalin, and stained with 0.5% Crystal Violet solution. Images were captured with an Olympus IX70 microscope. Assays were performed at least three times.

### Wound healing assay

To investigate rapamycin-induced inhibition of cell migration, a wound healing assay (or *in vitro* scratch assay) was performed as previously reported [40]. The wound healing assay is an easy, low-cost and well-developed method to measure cell migration *in vitro*. Cells were seeded into 6-well plates (Fisher Scientific, 353046) and grown to confluence. The vertical and horizontal cross-shaped scratch was made using a 1000-μL pipette tip on the monolayer of cells. The cells were washed three times with PBS, and the center of the cross, where the two scratch lines meet, was used to position the center of the wound gap. Fresh medium containing 1 ng/mL rapamycin or vehicle was added, and images of three fields, with a centrally located intersection of scratched lines, were taken using an upright microscope (Olympus IX71). After incubating for 24 h (or at the indicated time), the same fields were photographed again. The relative migration at an indicated time point was calculated by comparing the areas of the wounds at the initial time points. Scratched areas were quantified using the NIH ImageJ software (Bethesda, MD). The percentage of wound healing was expressed according to the following formula: [(initial scratched area, rapamycin added) – (resulting scratched area, rapamycin added)]/[(initial scratched area, vehicle added) – (resulting scratched area, vehicle added)] × 100.

### Boyden chamber-based cell migration and invasion assays

The *in vitro* migration and invasion assays were performed and modified using 8-μm pore-sized cell culture inserts (Falcon, 08-771-21) as previously reported [27]. The wells of a 24-well plate (Falcon, 353047) were filled with medium containing 10% FBS, with either rapamycin or vehicle, prior to loading the cells into the cell culture insert. Cells were starved overnight and added to serum-free medium, containing either rapamycin or vehicle, and loaded into the cell culture insert at a concentration of 2 × 10^4^ cells/well. For the invasion assay, the membrane at the bottom of the insert was coated with a layer of 0.2 mg/mL Matrigel (Fisher Scientific, CB354248). Cells were added to the upper compartment of the chamber, while the lower compartment was filled with culture medium. After 24 h, the cells were fixed in 10% formalin and stained with Crystal Violet (Fisher Scientific, C581-25). The cells remaining on the upper surface of the insert were removed with a Q-tip, and the plate with inserts was then imaged. The relative number of cells that invaded the gel barrier and passed through the insert’s pores were quantified by measuring the OD of the stain after removing the dye from the cells with 33% acetic acid (Fisher Scientific, A38S-212). The plate was read on a Cytation3 at a wavelength of 570 nm.

### Osteoblast and adipocyte differentiation assays

Cells were seeded into a 12-well plate (Fisher Scientific, 353043), cultured to 90−95% confluence, and treated with either osteogenic or adipogenic media. The osteoblast differentiation (OB diff) medium contained 50 μM ascorbic acid (Cayman Chemical, 16457), 100 nM Dexamethasone (Cayman Chemical, 11015), and 10 mM β-glycerophosphate (BGP) (Cayman Chemical, 14405). SJSA-1 and MG-63 were used as positive controls, and U-2 OS as a negative control. The medium was changed every three days, and the cells were stained on day 14. Cells were washed, fixed in 10% formalin, and then stained with Alizarin Red S (Sigma, A5533-25G). Images were taken with an Olympus IX70 microscope. Bone morphogenetic protein-2 (BMP2) (R&D Systems, 355-BM-010), a strong osteogenic inducer, was added to later assays at 100 ng/mL. The C2C12 cell line was used as the positive control. The adipocyte differentiation (Adi diff) medium contained 1 μl/mL of 125× 3-isobutyl-1-methylxanthine (Sigma, I5879-100MG), 1 mM dexamethasone, 10 mg/mL insulin (Cayman Chemical, 11015), and 1 mM rosiglitazone (Cayman Chemical, 11884). At day 0, the adipogenic medium contained all four reagents, and at day 4 it contained only insulin and rosiglitazone. After 7 days the cells were washed with PBS and fixed with 10% formalin, the lipid droplets were stained with 0.5% Oil Red O (Sigma, O0625-25G), and the cells were counterstained with Hematoxylin as needed. Images were then taken using an Olympus IX70 microscope. Differentiation was quantified by extracting Oil Red O with 50% of the well volume, i. e. 1 mL for a 6-well plate. The OD was measured with the Cytation3, using 200 μL of the extraction buffer, and absorbance was read at 492 nm. The C3H/10T1/2 cell line was used as a positive control.

### Cryosectioning and H&E staining

Collected fresh tumors from this study, or the original PDX tumors of OS-33, were embedded in O. C. T. compound (Tissue-Tek^®^). O. C. T. blocks were preserved at −80°C and sectioned with a LEICA CM 3050S cryostat into 8-μm thickness slices. The slides were fixed for 5 min using 10% formalin, and then washed for 1 min using tap water. Samples were stained with Hematoxylin for 1.5 min, and then washed again. Slides were immersed in bluing solution for 30 sec before rinsing three times in distilled water. Next, the slides were stained with Eosin Y solution for 20 sec and dehydrated with two changes of 95% ethanol solution, followed by two changes of 100% ethanol solution. Slides were then cleared in xylene, and a cover slip was applied. Slides were finally imaged using an Olympus IX70 microscope.

### Cytogenetics analysis, *TP53* mutation screening, gene fusion and circos plot analysis

The karyotypes for both P5 and P30 passage cells were produced according to a standard protocol, and the cytogenetic characterization was conducted by the Cytogenetics Laboratory of Sanford Medical Genetics (Sioux Falls, SD). Cells were exposed to 0.05 mg/mL colcemid for 6 h, incubated in 0.075 M KCl solution at 37°C for 40 min, and fixed with a mixture of methanol and glacial acetic acid (3:1, v/v). Drops of cell suspension were placed on cold slides and stained with the Remel Giemesa Plus Stain kit (Thermo Fisher Scientific) for 10 min. Samples were analyzed by trypsin Giemsa-banding, and results were expressed according to the recommendations of the International System for Human Cytogenetic Nomenclature (ISCN, 2016). To screen for *TP53* mutations, genomic DNA was extracted from ~2 million cells using the DNeasy Blood and Tissue kit (Qiagen, Valencia, CA, USA), according to the manufacturer’s instructions. PCR was performed using AccuStart™ II GelTrack PCR SuperMix (QuantaBio), and the primers used were: TP53E8&9For, TTAAATGGGACAGGTAGGAC (2888-2907, NCBI Accession: AH007667.2); TP53E8&9Rev (3324-3344, NCBI Accession: AH007667.2), TTGCAGGTAAAACAGTCAAGA; NOTCH2E34For, GCCTCACCCAACCCTATGTT (158464-158483, NCBI Accession: NG_008163.1); NOTCH2 E34Rev TAGGCTGGGAGAATGGTCTGA (159030-159050, NCBI Accession: NG_008163.1). Sanger sequencing of the PCR products obtained from genomic DNAs was performed by Eurofins Genomics. Fusion events of the OS-33 PDX model were previously identified using multiple fusion-calling tools (STAR-Fusion v1.1.0, FusionCatcher v0.99.7b, deFuse and SOAPFuse) [4]. Additional details about the filtering and annotation of the fusions can be found elsewhere [4]. The Circos plot was generated with circus [41]. The Y chromosome was dropped from the plot; fusions with partners from two distinct strands were labeled as blue, otherwise as red.

### Statistical analysis

Reported values are expressed as means ± standard deviation. Data were analyzed, where appropriate, using the Student’s *t*-test (comparing two groups only), or analysis of variance (ANOVA) with the Tuckey HSD test for multiple comparisons. *p*-values < 0.05 were considered statistically significant.

## SUPPLEMENTARY MATERIALS





## Abbreviations

OS: Osteosarcoma
PDX: patient-derived xenografts
PPTC: Pediatric Preclinical Testing Consortium
mTOR: mammalian target of rapamycin
BMP2: Bone morphogenetic protein 2
CCK-8: Cell Counting Kit-8
PCR: polymerase chain reaction
TP53: tumor protein p53
OB diff: osteoblast differentiation
Adi diff: adipocyte differentiation
