# Supplementary material for: Development and characterization of the novel human osteosarcoma cell line COS-33 with sustained activation of the mTOR pathway
Source: Oncotarget. 2020 Jul 7;11(27):2597–610. doi: 10.18632/oncotarget.27611 (PMC7343631; doi:10.18632/oncotarget.27611)
Supplement: Supplementary file 1 [file oncotarget-11-2597-s001.pdf]

Development and characterization of the novel human osteosarcoma cell line COS-33 with sustained activation of the mTOR pathway

SUPPLEMENTARY MATERIALS

Supplementary Table 1: High confidence fusions. See Supplementary Table 1
